# Mathematical modeling and estimation of physicochemical characteristics of pneumonia treatment drugs through a novel approach *K*-Banhatti topological descriptors

**DOI:** 10.3389/fchem.2025.1564809

**Published:** 2025-05-02

**Authors:** Abdul Rauf Khan, Ifra Naeem, Fairouz Tchier, Fikadu Tesgera Tolasa, Shahid Hussain

**Affiliations:** ^1^ Department of Mathematics, Faculty of Sciences, Ghazi University, Dera Ghazi Khan, Pakistan; ^2^ Mathematics Department, College of Science, King Saud University, Riyad, Saudi Arabia; ^3^ Department of Mathematics, Dambidollo University, Oromia, Ethiopia; ^4^ Energy Engineering Division, Department of Engineering Science and Mathematics, Lulea University of Technology, Lulea, Sweden

**Keywords:** molecular structure, anti-pneumonia drugs, physicochemical properties, topological descriptors, K-Banhatti descriptors, regression models, QSPR testing, chemical graph theory

## Abstract

**Introduction:**

Pneumonia is the primary cause of mortality in preterm infants in developing nations; yet, early detection and treatment can significantly reduce mortality rates. Pharmaceutical researchers are diligently striving to identify avariety of drugs that might effectively cure pneumonia.

**Method:**

We are motivated to examine the quantitative structureproperty relationships (QSPR) of anti-pneumonia pharmaceuticals. We employed *K*-Banhatti topological descriptors and analyzed the findings to achieve this. For estimation of physicochemical properties of pneumonia treatment drugs we utilized linear, quadratic, cubic, and biquadratic regression analyses.

**Results and Conclusion:**

The drugs comprise linezolid, ceftabiprole, and clarithromycin, among others. Topological descriptors enable the exploration of the complexity, connectivity, and other essential attributes of molecules. The quantitative structure-property relationship (QSPR) analysis of pharmaceuticals for illness treatment employing *K*-Banhatti topological descriptors is an economical approach utilised by pharmaceutical researchers. We performed a QSPR analysis on 20 anti-pneumonia drugs to ascertain the most precise predictions for five properties: enthalpy, flash point, molecular weight, molar volume, and molar refractivity, employing five *K*-Banhatti indices. To do this, we used linear, quadratic, cubic, and biquadratic regression analyses to find links between molecules and the physical and chemical properties of drugs used to treat pneumonia. Employing molecular descriptors and regression models to investigate chemical patterns is a cost-effective and theoretical methodology.

## 1 Introduction

Pneumonia is an infectious disease and is frequently induced by bacterial, viral, or fungal infections that specifically affect the lungs, leading to inflammation of the alveoli (Scotta et al., 2019). Common bacterial agents include *Streptococcus* pneumoniae and *Haemophilus* influenzae, while viral agents may include the influenza virus, respiratory syncytial virus (RSV), and coronaviruses (Marangu and Zar, 2019). Pneumonia transmission occurs via inhalation of airborne droplets from coughs or sneezes, direct contact with infected individuals, or by touching contaminated objects and then contacting the face.

Each year, around two million children under 5 years old succumb to pneumonia in developing countries, primarily due to infections caused by *streptococcus* or the influenza virus (Singh and Aneja, 2011; Leung et al., 2018). Pneumonia ranks among the primary causes of mortality and morbidity in children globally. Pneumonia is a sudden respiratory infection caused by various organisms, impacting management strategies in the developing world (Shann, 1995). Lungs exhibit swelling of the airway sacs and pleural effusion, which occurs when the lung is infiltrated with fluid. Pneumonia impacts 10 to 15 percent of children with respiratory issues. Underdeveloped and rising nations are predisposed to elevated pneumonia rates due to factors such as overcrowding, pollution, unsanitary environmental conditions, and restricted access to healthcare (Wojsyk-Banaszak and Breeborowicz, 2013; Wardlaw et al., 2006).

Pneumonia in toddlers under the age of two is especially perilous. The lack of adequate immunizations and limited access to healthcare services in several impoverished communities in developing and underdeveloped nations results in undetected pneumonia, thereby exacerbating respiratory conditions (Rudan et al., 2004). Over the past 10 years, the number of cases and severity of pneumonia in children, as well as their death rates, have gone down significantly. This is because the economy is better, care is better, more effective treatment and prevention strategies are used, and more vaccinations are made available, especially the combination vaccines against pneumococcal disease (PCV) and hepatitis B. Survival rates have markedly improved since the 20th century due to advancements in immunisations and pharmaceuticals (Madhi et al., 2008; Scelfo et al., 2021). Moreover, increasing evidence associates childhood pneumonia and lower respiratory tract infections (LRTIs) with diminished lung capacity in early childhood and an escalation of long-term, latent respiratory conditions in both children and adults, such as asthma and chronic bronchitis (Munywoki et al., 2013). The illness can be classified based on its origin, such as community-acquired or hospital-acquired pneumonia (Venditti et al., 2009).

Pneumonia is treatable with various medications. Potentially appropriate medications include beta-lactams such as penicillin and amoxicillin in combination with a macrolide, or fluoroquinolone antibiotics like Levaquin (Garau, 2005). Macrolide antibiotics, including tetracycline, azithromycin, and clarithromycin, may serve as initial treatment options (Alvarez-Elcoro and Enzler, 1999; Sood, 1999). Adverse events associated with ceftobiprole in patients indicated that the medication demonstrated an acceptable safety profile (Liapikou et al., 2015).

**FIGURE 1:**
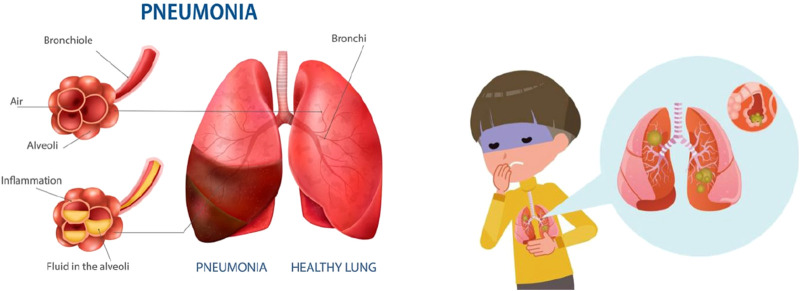
Pneumonia in children.

Despite the discovery of antibiotics, the prevalence of pneumonia has likely remained relatively stable over the past century; however, the overall mortality rate has significantly decreased. Determining the responsible pathogen may present challenges. Diagnosis may be confirmed through blood tests, sputum culture, and chest X-rays (Parveen and Sathik, 2011). Symptoms and a physical examination are commonly employed to establish a diagnosis. Potential symptoms include (Harari et al., 1991): Expectoration of greenish or yellow mucus, or potentially bloody mucus, may occur. Productive cough with phlegm production, Dyspnoea, Fever and anorexia. Figure 1 shows the pneumonia infection.

According to the chemical theory of graphs, atoms are represented as vertices of a graph, and the bonds that bind them together are described as edges (Khan et al., 2024a). A graph G is characterized as a pair of E is a set of connected vertices with components known as edges, and V is a collection of items known as vertices (Khan et al., 2023a). The degree of any vertex σ , denoted by the symbol $\hat{d}\left(\sigma \right)$ , is expressed as the number of vertices that are close to it. One type of structural descriptor that may be determined using a chemical substance’s molecule network is a topological indicator (Dehmer et al., 2010; Khan et al., 2025). Chemical graph theory is a branch of graph theory that combines chemical models mathematically (Khan et al., 2024b). It emphasizes topological aspects that are directly associated with molecular chemical properties (Galvez et al., 2011).

A topological descriptor is a distinctive number that characterizes the intrinsic structure of the molecular graph. In QSPR and QSAR, scientists use numerical parameters from a chemical graph network. Its uses are increasing in medication design. Wiener pioneered the concept of topological descriptors with the distance base topological descriptor (Hayat, 2017). Husin et al. (2015) discuss Zagreb polynomials and topological indices for a synthesized molecule composed of branched units known as monomers. In Vijay et al. (2023), the study focused on the vertex version of the distance-based topological indices, the entropy of the topological indices and their numerical analysis of aluminophosphates. Fathi et al. (2024) examined topological indices based on valency, induced by quantitative structural relationships, to predict the structural properties of Ni tetrathiafulvalene tetrathionate (NiTTFtt) in a 2D sheet configuration.

A molecular structure’s topological index gives numerical values that are useful for property prediction. Topological descriptors are useful tools for researchers who want to figure out the different topological properties of drugs (Hakami et al., 2024), networks (Chu et al., 2023; Khan et al., 2024c), and materials (Imran et al., 2023; Khan et al., 2023b). Numerous researchers have investigated different topological descriptors of material-related networks in Hakami et al. (2025), Khan et al. (2024d), Khan et al. (2024e) and the estimation of physical and chemical properties of various drugs in Husin et al. (2024). Nadeem et al. put forward the QSPR idea on babesiosis drugs (Awan et al., 2025) and antimalarial compounds modeling results depict the clear picture (Awan et al., 2024) said disease efficiently. Fozia made a great contribution to cardiac (Bashir Farooq et al., 2022) drugs and blood cancer (Nasir et al., 2022) and Sobia done QSPR application of infertility Drugs (Sultana, 2023) modeling is done.

## 2 Material and methodology

**FIGURE 2:**
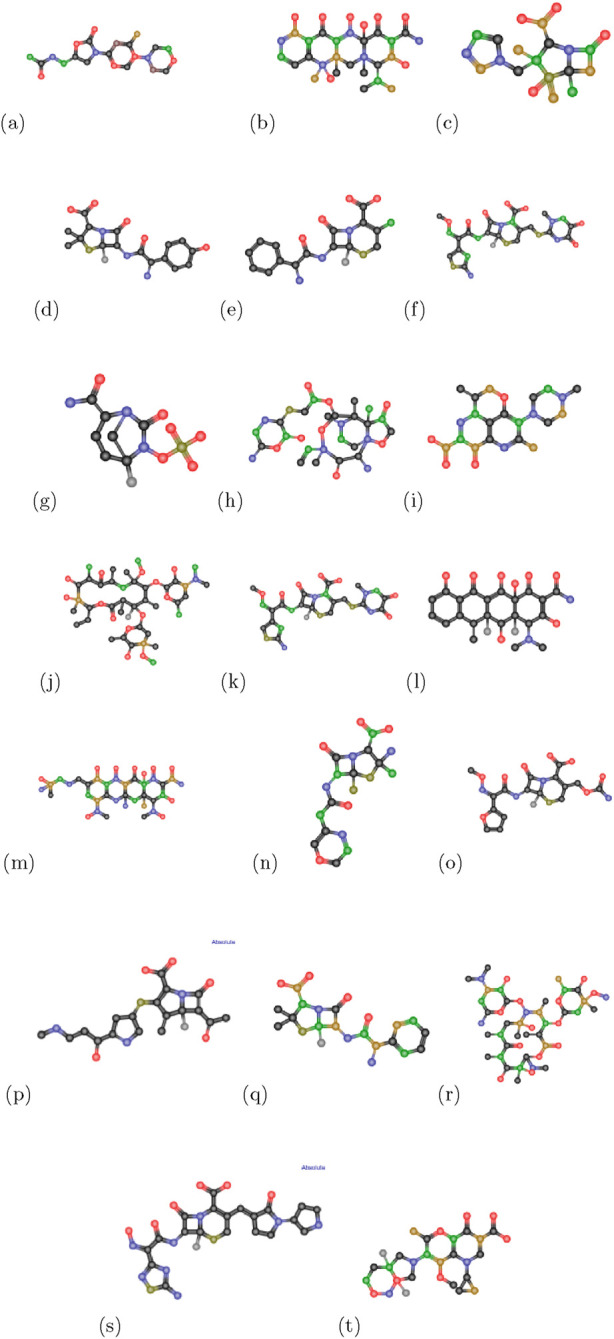
Chemical structures of anti-pneumonia drugs **(a)** linezolid **(b)** tetracycline **(c)** tazobactam **(d)** amoxicillin **(e)** cefaclor **(f)** ceftriaxone **(g)** avibactam **(h)** lefamulin **(i)** levaquine **(j)** clarithromycin **(k)** cefpodoxime **(l)** doxycycline **(m)** omadacycline **(n)** penicillin **(o)** cefuroxime **(p)** carbapenem **(q)** unasyn **(r)** erythromycin **(s)** ceftabiprole **(t)** moxifloxacin.

The current study examines the following anti-pneumonia drugs: Linezolid $\left(C_{16}H_{20}FN_{3}O_{4}\right)$ , Tetracycline $\left(C_{22}H_{24}N_{2}O_{8}\right)$ , Tazobactam $\left(C_{10}H_{12}N_{4}O_{5}S\right)$ , Amoxicillin $\left(C_{16}H_{19}N_{3}O_{5}S\right)$ , Cefaclor $\left(C_{15}H_{14}ClN_{3}O_{4}S\right)$ , Ceftriaxone $\left(C_{18}H_{18}N_{8}O_{7}S_{3}\right)$ , Avibactam $\left(C_{7}H_{11}N_{3}O_{6}S\right)$ , Lefamulin $\left(C_{28}H_{45}NO_{5}S\right)$ , Clarithromycin $\left(C_{38}H_{69}NO_{13}\right)$ , Levaquin $\left(C_{18}H_{20}FN_{3}O_{4}\right)$ Cefpodoxime $\left(C_{15}H_{17}N_{5}O_{6}S_{2}\right)$ , Doxycycline $\left(C_{22}H_{24}N_{2}O_{8}\right)$ , Omadacycline $\left(C_{29}H_{40}N_{4}O_{7}\right)$ , Penicillin $\left(C_{16}H_{17}N_{2}NaO_{4}S\right)$ , Cefuroxime $\left(C_{16}H_{16}N_{4}O_{8}S\right)$ , Carbapenem $\left(C_{18}H_{29}N_{3}O_{5}S\right)$ , Erythromycin $\left(C_{37}H_{67}NO_{13}\right)$ , Ceftobiprole $\left(C_{20}H_{22}N_{8}O_{6}S_{2}\right)$ , Moxifloxacin $\left(C_{21}H_{24}FN_{3}O_{4}S\right)$ and Unasyn $\left(C_{16}H_{19}N_{8}O_{6}S\right)$ are examined. Additionally, we sourced the drugs, their chemical formulas, and their physical and chemical properties from https://www.chemicalbook.com/. In this study, anti-pneumonia medications are shown by plain graphs in Figure 2. The drug’s topological indices are computed using vertex division, edge division, and edge degree algorithms. The degree of edge $\overset{˘}{\overset{˘}{e}}=\sigma \gamma$ is represented by $R\left(\overset{˘}{\overset{˘}{e}}\right)$ and

$$R\left(\overset{˘}{\overset{˘}{e}}\right)=R\left(\sigma \right)+R\left(\gamma \right)-2$$

The graph’s greatest and lowest degree can be expressed by Δ(M) and δ(m) . Equations 1–5 offer formulae for descriptors which will be used in the sequel.

The first K -Banhatti descriptor is defined as (Mahboob et al., 2022)

$$B_{1}\left(G\right)=\underset{I\in E\left(G\right)}{\sum }R\left(I\right)+R\left(\overset{˘}{\overset{˘}{e}}\right)$$ (1)

The second K -Banhatti descriptor is defined as (Ghani et al., 2022)

$$B_{2}\left(G\right)=\underset{I\in E\left(G\right)}{\sum }R\left(I\right)\;*\;R\left(\overset{˘}{\overset{˘}{e}}\right)$$ (2)

The first Hyper K -Banhatti descriptor (Kulli, 2016)

$${HB}_{1}\left(G\right)=\underset{I\in E\left(G\right)}{\sum }{\left[R\left(I\right)+R\left(\overset{˘}{\overset{˘}{e}}\right.\right]}^{2}$$ (3)

The second Hyper K -Banhatti descriptor (Almalki and Tabassum, 2024)

$${HB}_{2}\left(G\right)=\underset{I\in E\left(G\right)}{\sum }{\left[R\left(I\right)\;*\;R\left(\overset{˘}{\overset{˘}{e}}\right)\right]}^{2}$$ (4)

The K -Banhatti Harmonic descriptor (Anjum and Safdar, 2019)

$$H_{b}\left(G\right)=\underset{I\in E\left(G\right)}{\sum }\frac{2}{\left[R\left(I\right)+R\left(\overset{˘}{\overset{˘}{e}}\right)\right]}$$ (5)

## 3 Results

In this study, we compute topological descriptor values using two-dimensional graphs of pneumonia treatment drugs. We used various methods, including edge dividing, vertex degree evaluation, and edge degree methodology, to calculate the K -Banhatti descriptors. Edge division and edge degree of Linezolid are presented in Table 1. K -Banhatti descriptors may be calculated as indicated below.

**TABLE 1:** Edge division and edge degree of Linezolid.

Edge division	${\tilde{E}}_{1,3}$	${\tilde{E}}_{2,2}$	${\tilde{E}}_{2,3}$	${\tilde{E}}_{3,3}$
Edge degree	2	2	3	4
Cardinality	4	6	12	4

### 3.1 K -Banhatti descriptors for linezolid $\left(C_{16}H_{20}FN_{3}O_{4}S\right)$

The following linezolid results were obtained by utilizing Equations 1–5; Table 1.

• First K -Banhatti descriptor

$$\begin{array}{ll} B_{1}\left(G\right) & =\underset{I\in E\left(G\right)}{\sum }R\left(I\right)+R\left(\overset{˘}{\overset{˘}{e}}\right)\\ & =4\left(3+5\right)+6\left(4+4\right)+12\left(5+6\right)2+4\left(7+7\right)\\ & =268 \end{array}$$

• Second K -Banhatti descriptor

$$\begin{array}{ll} B_{2}\left(G\right) & =\underset{I\in E\left(G\right)}{\sum }R\left(I\right)\;*\;R\left(\overset{˘}{\overset{˘}{e}}\right)\\ & =4\left(2+6\right)+6\left(4+4\right)+12\left(6+9\right)+4\left(12+12\right)\\ & =356 \end{array}$$

• First Hyper K -Banhatti descriptor

$$\begin{array}{ll} {HB}_{1}\left(G\right) & =\underset{I\in E\left(G\right)}{\sum }{\left[R\left(I\right)+R\left(\overset{˘}{\overset{˘}{e}}\right.\right]}^{2}\\ & =4\left(9+25\right)+6\left(16+16\right)+12\left(25+36\right)2+4\left(49+49\right)\\ & =1452 \end{array}$$

• Second Hyper K -Banhatti descriptor

$$\begin{array}{ll} {HB}_{2}\left(G\right) & =\underset{I\in E\left(G\right)}{\sum }{\left[R\left(I\right)\;*\;R\left(\overset{˘}{\overset{˘}{e}}\right)\right]}^{2}\\ & =4\left(4+36\right)+6\left(16+16\right)+12\left(36+81\right)2+4\left(144+144\right)\\ & =2908 \end{array}$$

• Harmonic K -Banhatti descriptor

$$\begin{array}{ll} H_{b}\left(G\right) & =\underset{I\in E\left(G\right)}{\sum }\frac{2}{\left[R\left(I\right)+R\left(\overset{˘}{\overset{˘}{e}}\right)\right]}\\ & =4\left(2/3+2/5\right)+6\left(2/4+2/4\right)+12\left(2/5+2/6\right)2+4\left(2/7+2/7\right)=21.364 \end{array}$$

### 3.2 K -Banhatti descriptors for Unasyn $\left(C_{16}H_{19}N_{3}O_{4}S\right)$

The following Unasyn results were obtained by utilizing Equations 1–5; Table 2.

$$\begin{array}{ll} B_{1}\left(G\right) & =\underset{I\in E\left(G\right)}{\sum }R\left(I\right)+R\left(\overset{˘}{\overset{˘}{e}}\right)\\ & =5\left(3+5\right)+3\left(4+7\right)+4\left(4+4\right)+4\left(5+6\right)+6\left(7+7\right)+2\left(6+8\right)+3\left(8+9\right)=312 \end{array}$$

$$\begin{array}{ll} B_{2}\left(G\right) & =\underset{I\in E\left(G\right)}{\sum }R\left(I\right)\;*\;R\left(\overset{˘}{\overset{˘}{e}}\right)\\ & =5\left(2+6\right)+3\left(3+12\right)+4\left(4+4\right)+4\left(6+9\right)+6\left(12+12\right)+2\left(8+16\right)+3\left(15+20\right)=474 \end{array}$$

$$\begin{array}{ll} {HB}_{1}\left(G\right) & =\underset{I\in E\left(G\right)}{\sum }{\left[R\left(I\right)+R\left(\overset{˘}{\overset{˘}{e}}\right.\right]}^{2}\\ & =5\left(9+25\right)+3\left(16+49\right)+4\left(16+16\right)+4\left(25+36\right)+6\left(49+49\right)+2\left(36+64\right)+3\left(64+81\right)=1960 \end{array}$$

$$\begin{array}{ll} {HB}_{2}\left(G\right) & =\underset{I\in E\left(G\right)}{\sum }{\left[R\left(I\right)\;*\;R\left(\overset{˘}{\overset{˘}{e}}\right)\right]}^{2}\\ & =5\left(4+36\right)+3\left(9+144\right)+4\left(16+16\right)+4\left(36+81\right)+6\left(144+144\right)+2\left(64+256\right)+3\left(225+400\right)=5498 \end{array}$$

$$\begin{array}{ll} H_{b}\left(G\right) & =\underset{I\in E\left(G\right)}{\sum }\frac{2}{\left[R\left(I\right)+R\left(\overset{˘}{\overset{˘}{e}}\right)\right]}\\ & =5\left(2/3+2/5\right)+3\left(2/4+2/7\right)+4\left(2/4+2/4\right)+4\left(2/5+2/6\right)+6\left(2/7+2/7\right)+2\left(2/6+2/8\right)+3\left(2/8+2/9\right)=20.6357 \end{array}$$

**TABLE 2:** Edge division and edge degree of Unasyn.

Edge division	${\tilde{E}}_{1,3}$	${\tilde{E}}_{1,4}$	${\tilde{E}}_{2,2}$	${\tilde{E}}_{2,3}$	${\tilde{E}}_{3,3}$	${\tilde{E}}_{2,4}$	${\tilde{E}}_{3,4}$
Edge degree	2	3	2	3	4	4	5
Cardinality	5	3	4	4	6	2	3

### 3.3 K -Banhatti descriptors for Cefuroxime $\left(C_{16}H_{16}N_{4}O_{8}S\right)$

The following Cefuroxime results were obtained by utilizing Equations 1–5; Table 3

$$\begin{array}{ll} B_{1}\left(G\right) & =\underset{I\in E\left(G\right)}{\sum }R\left(I\right)+R\left(\overset{˘}{\overset{˘}{e}}\right)\\ & =1\left(2+3\right)+6\left(3+5\right)+6\left(4+4\right)+10\left(5+6\right)+5\left(7+7\right)+2\left(6+8\right)+1\left(8+9\right)1\left(4+7\right)+=337 \end{array}$$

$$\begin{array}{ll} B_{2}\left(G\right) & =\underset{I\in E\left(G\right)}{\sum }R\left(I\right)\;*\;R\left(\overset{˘}{\overset{˘}{e}}\right)\\ & =1\left(1+2\right)+6\left(2+6\right)+6\left(4+4\right)+10\left(6+9\right)+5\left(12+12\right)+2\left(8+16\right)+1\left(15+20\right)+1\left(3+12\right)=466 \end{array}$$

$$\begin{array}{ll} {HB}_{1}\left(G\right) & =\underset{I\in E\left(G\right)}{\sum }{\left[R\left(I\right)+R\left(\overset{˘}{\overset{˘}{e}}\right.\right]}^{2}\\ & =1\left(4+9\right)+6\left(9+25\right)+6\left(16+16\right)+10\left(25+36\right)+5\left(49+49\right)+2\left(36+64\right)+1\left(64+81\right)1\left(16+49\right)=1919 \end{array}$$

$$\begin{array}{ll} {HB}_{2}\left(G\right) & =\underset{I\in E\left(G\right)}{\sum }{\left[R\left(I\right)\;*\;R\left(\overset{˘}{\overset{˘}{e}}\right)\right]}^{2}\\ & =1\left(1+4\right)+6\left(4+36\right)+6\left(16+16\right)+10\left(36+81\right)+5\left(144+144\right)+2\left(64+256\right)+1\left(225+400\right)+1\left(9+144\right)=4465 \end{array}$$

$$\begin{array}{lll} H_{b}\left(G\right) & =\underset{I\in E\left(G\right)}{\sum }\frac{2}{\left[R\left(I\right)+R\left(\overset{˘}{\overset{˘}{e}}\right)\right]} & \\ & =1\left(2/2+2/3\right)+6\left(2/3+2/5\right)+6\left(2/4+2/4\right)+10\left(2/5+2/6\right)+5\left(2/7+2/7\right)+2\left(2/6+2/8\right)+1\left(2/8+2/9\right)1\left(2/4+2/7\right)=26.6957 \end{array}$$

**TABLE 3:** Edge division and edge degree of Cefuroxime.

Edge division	${\tilde{E}}_{1,2}$	${\tilde{E}}_{1,3}$	${\tilde{E}}_{2,2}$	${\tilde{E}}_{2,3}$	${\tilde{E}}_{3,3}$	${\tilde{E}}_{2,4}$	${\tilde{E}}_{3,4}$	${\tilde{E}}_{1,4}$
Edge degree	1	2	2	3	4	4	5	3
Cardinality	1	6	6	10	5	2	1	1

### 3.4 K -Banhatti descriptors for Avibactam $\left(C_{7}H_{11}N_{3}O_{6}S\right)$

The following Avibactam results were obtained by utilizing Equations 1–5; Table 4.

$$\begin{array}{ll} B_{1}\left(G\right) & =\underset{I\in E\left(G\right)}{\sum }R\left(I\right)+R\left(\overset{˘}{\overset{˘}{e}}\right)\\ & =3\left(3+5\right)+4\left(4+7\right)+1\left(4+4\right)+3\left(5+6\right)+4\left(7+7\right)+3\left(6+8\right)+1\left(8+9\right)=224 \end{array}$$

$$\begin{array}{ll} B_{2}\left(G\right) & =\underset{I\in E\left(G\right)}{\sum }R\left(I\right)\;*\;R\left(\overset{˘}{\overset{˘}{e}}\right)\\ & =3\left(2+6\right)+4\left(3+12\right)+1\left(4+4\right)+3\left(6+9\right)+4\left(12+12\right)+3\left(8+16\right)+1\left(15+20\right)=340 \end{array}$$

$$\begin{array}{ll} {HB}_{1}\left(G\right) & =\underset{I\in E\left(G\right)}{\sum }{\left[R\left(I\right)+R\left(\overset{˘}{\overset{˘}{e}}\right.\right]}^{2}\\ & =3\left(9+25\right)+4\left(16+49\right)+1\left(16+16\right)+3\left(25+36\right)+4\left(49+49\right)+3\left(36+64\right)+1\left(64+81\right)=1414 \end{array}$$

$$\begin{array}{ll} {HB}_{2}\left(G\right) & =\underset{I\in E\left(G\right)}{\sum }{\left[R\left(I\right)\;*\;R\left(\overset{˘}{\overset{˘}{e}}\right)\right]}^{2}\\ & =3\left(4+36\right)+4\left(9+144\right)+1\left(16+16\right)+3\left(36+81\right)+4\left(144+144\right)+3\left(64+256\right)+1\left(225+400\right)=3852 \end{array}$$

$$\begin{array}{ll} H_{b}\left(G\right) & =\underset{I\in E\left(G\right)}{\sum }\frac{2}{\left[R\left(I\right)+R\left(\overset{˘}{\overset{˘}{e}}\right)\right]}\\ & =3\left(2/3+2/5\right)+4\left(2/4+2/7\right)+1\left(2/4+2/4\right)+3\left(2/5+2/6\right)+4\left(2/7+2/7\right)+3\left(2/6+2/8\right)+1\left(2/8+2/9\right)=14.06 \end{array}$$

**TABLE 4:** Edge division and edge degree of Avibactam.

Edge division	${\tilde{E}}_{1,3}$	${\tilde{E}}_{1,4}$	${\tilde{E}}_{2,2}$	${\tilde{E}}_{2,3}$	${\tilde{E}}_{3,3}$	${\tilde{E}}_{2,4}$	${\tilde{E}}_{3,4}$
Edge degree	2	3	2	3	4	4	5
Cardinality	3	4	1	3	4	3	1

Remark 1. Other K -Banhatti topological descriptors of pneumonia treatment drugs are computed in a similar way as computed above, and their values are presented in Table 5.

**TABLE 5:** Computational values of K -Banhatti Descriptors of Anti-Pneumonia Drugs.

Drugs	$B_{1}$	$B_{2}$	${HB}_{1}$	${HB}_{2}$	$H_{b}$
Linezolid	268	356	1,452	2,908	21.364
Omadacycline	520	850	3,326	10,566	33.453
Moxifloxacin	406	616	2,536	6,964	26.569
Ceftriaxone	440	634	2,604	6,406	31.675
Unasyn	312	474	1,960	5,498	20.6357
Lefamulin	453	716	2,966	9,504	30.213
Carbapanem	336	500	2,058	4,282	24.055
Cefuroxime	337	466	1,919	4,465	26.696
Cefaclor	342	440	1,810	4,724	21.392
Tetracycline	458	752	3,122	10,372	27.054
Amoxicillin	326	496	2052	5,708	21.83
Ceftabiprole	454	658	2,698	6,678	37.376
Doxycycline	446	710	2,934	8,834	27.69
Avibactam	224	340	1,414	3,852	14.06
Tazobactam	280	454	1,886	6,354	16.911
Clarithromycin	626	944	3,912	10,880	43.984
Levaquin	322	462	1890	4,386	22.462
Erythromycin	598	886	3,666	9,678	46.817
Cefpodoxima	344	516	2,124	6,184	26.907
Pencillin	298	448	1,852	5,116	19.905

## 4 Quantitative structure-property relation analysis of anti-pneumonia drugs

**FIGURE 3:**
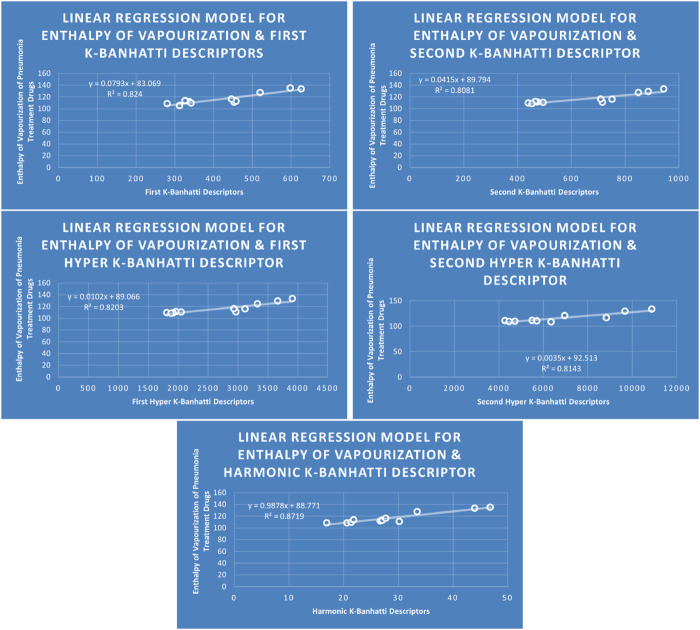
LR Models of Enthalpy of Vaporization for Pneumonia Treatment Drugs.

**FIGURE 4:**
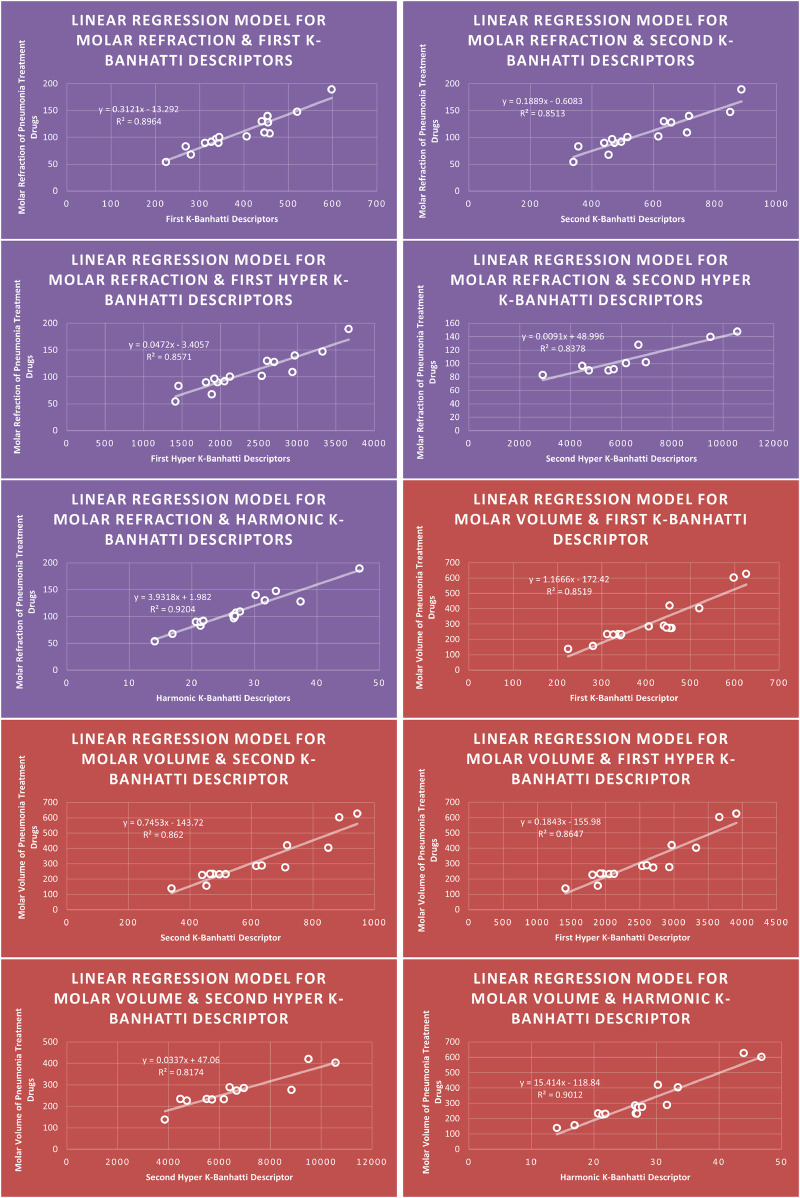
LR models of molar refraction and molar volume for pneumonia treatment drugs.

**FIGURE 5:**
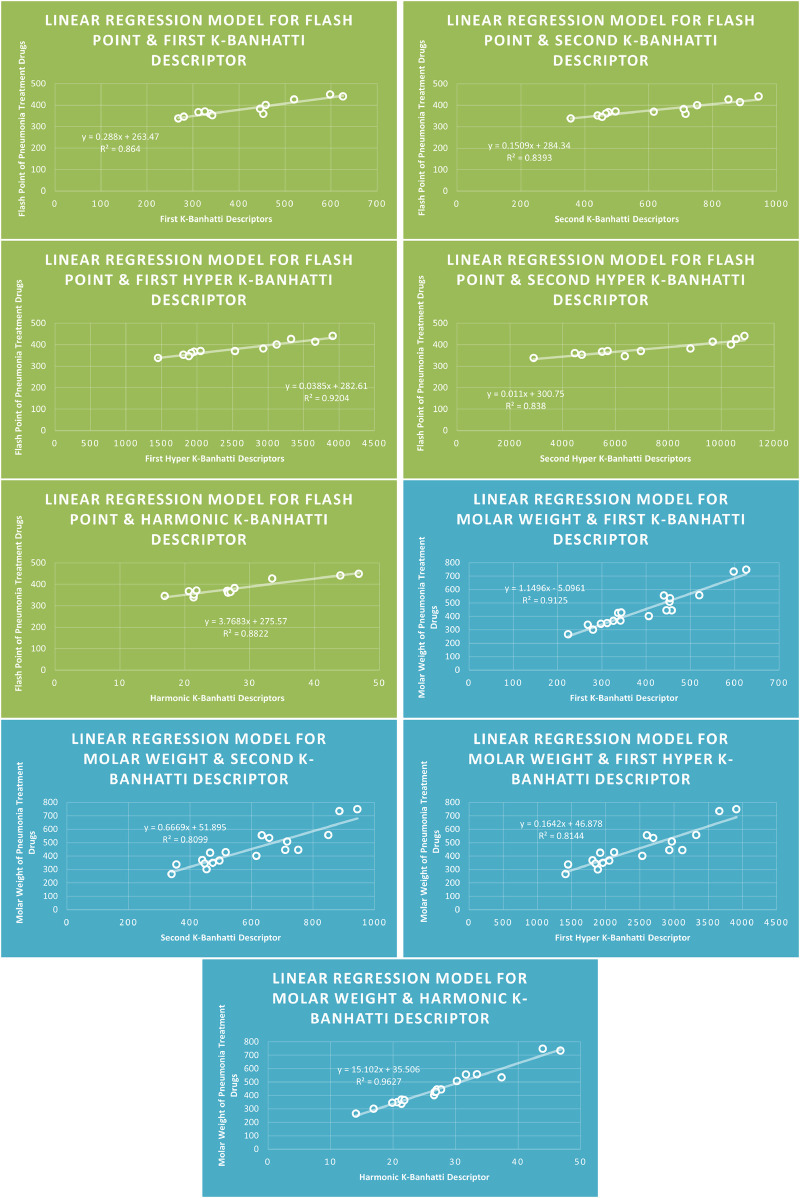
**LR** models of flash point and molar weight for pneumonia treatment drugs.

**FIGURE 6:**
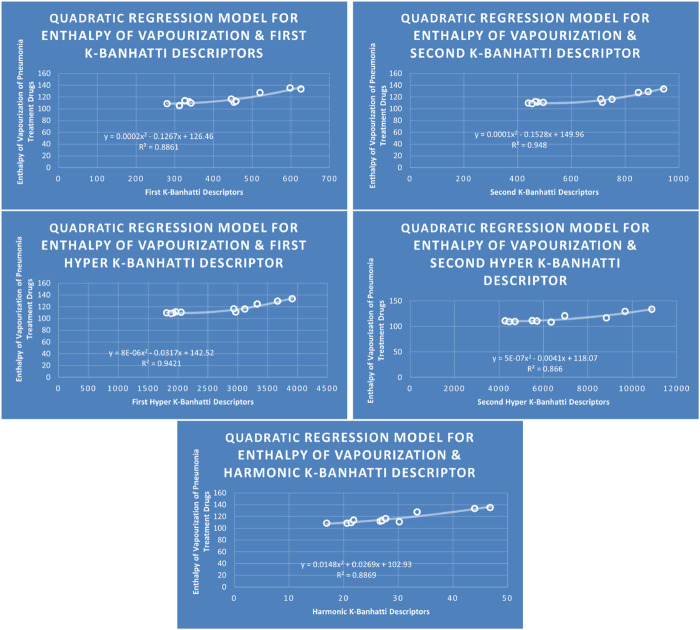
QR models of enthalpy of vaporization for pneumonia treatment drugs.

**FIGURE 7:**
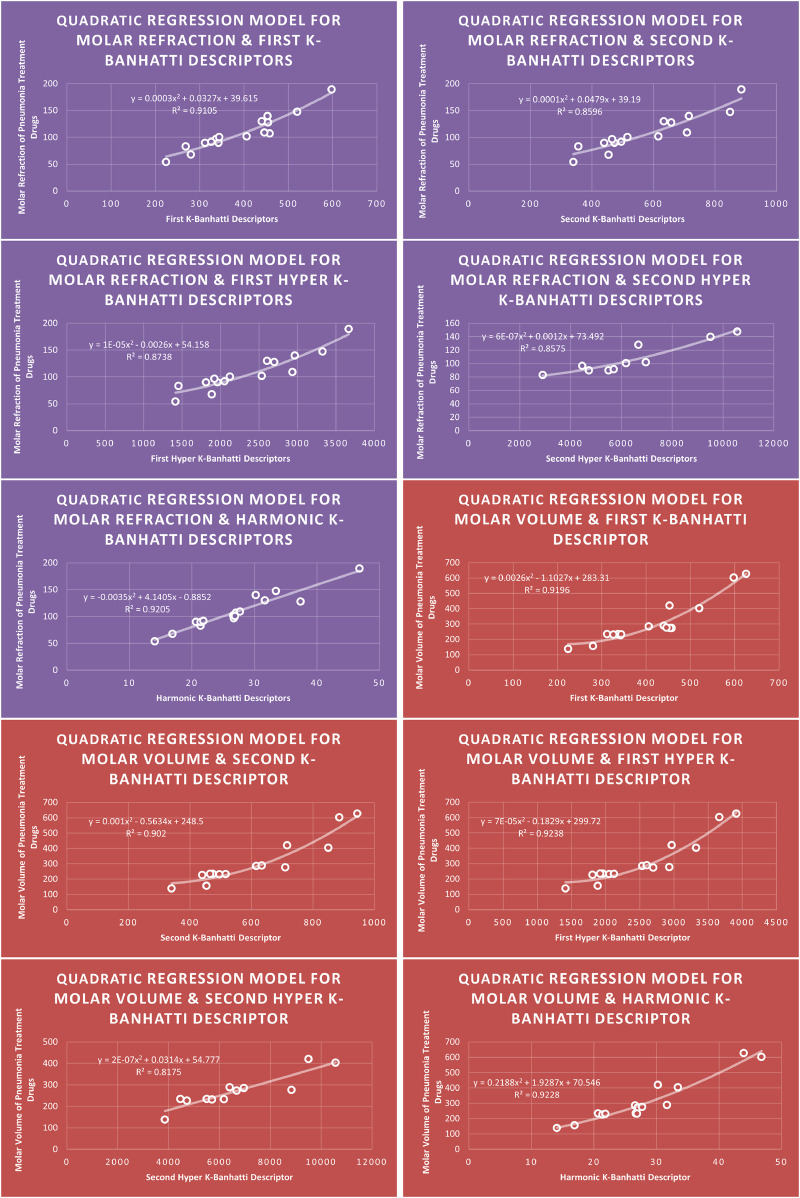
**QR** models of molar refraction and molar volume for pneumonia treatment drugs.

**FIGURE 8:**
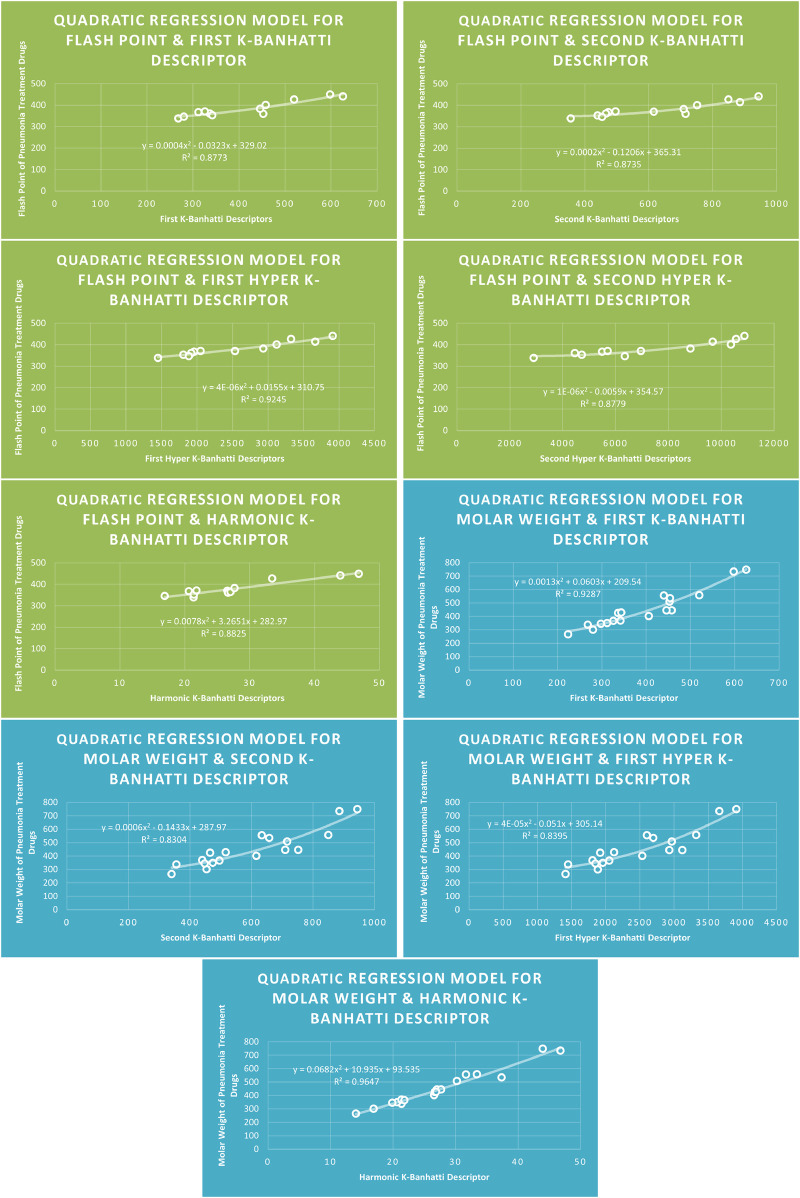
**QR** models of flash point and molar weight for pneumonia treatment drugs.

**FIGURE 9:**
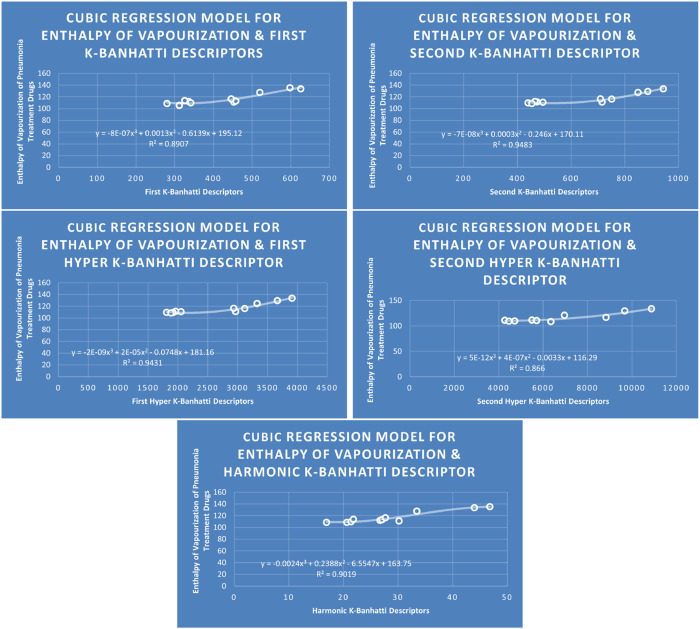
**CR** models of enthalpy of vapourization for pneumonia treatment drugs.

**FIGURE 10:**
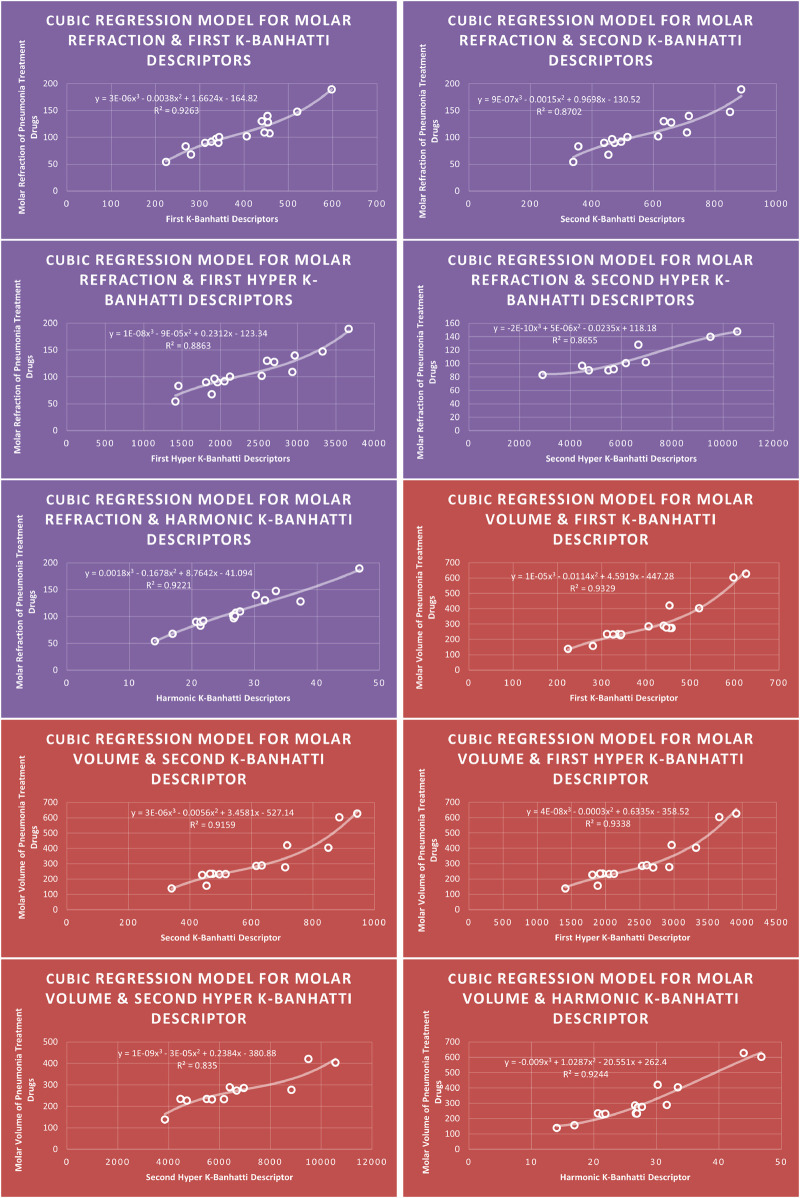
**CR** models of molar refraction and molar volume for pneumonia treatment drugs.

**FIGURE 11:**
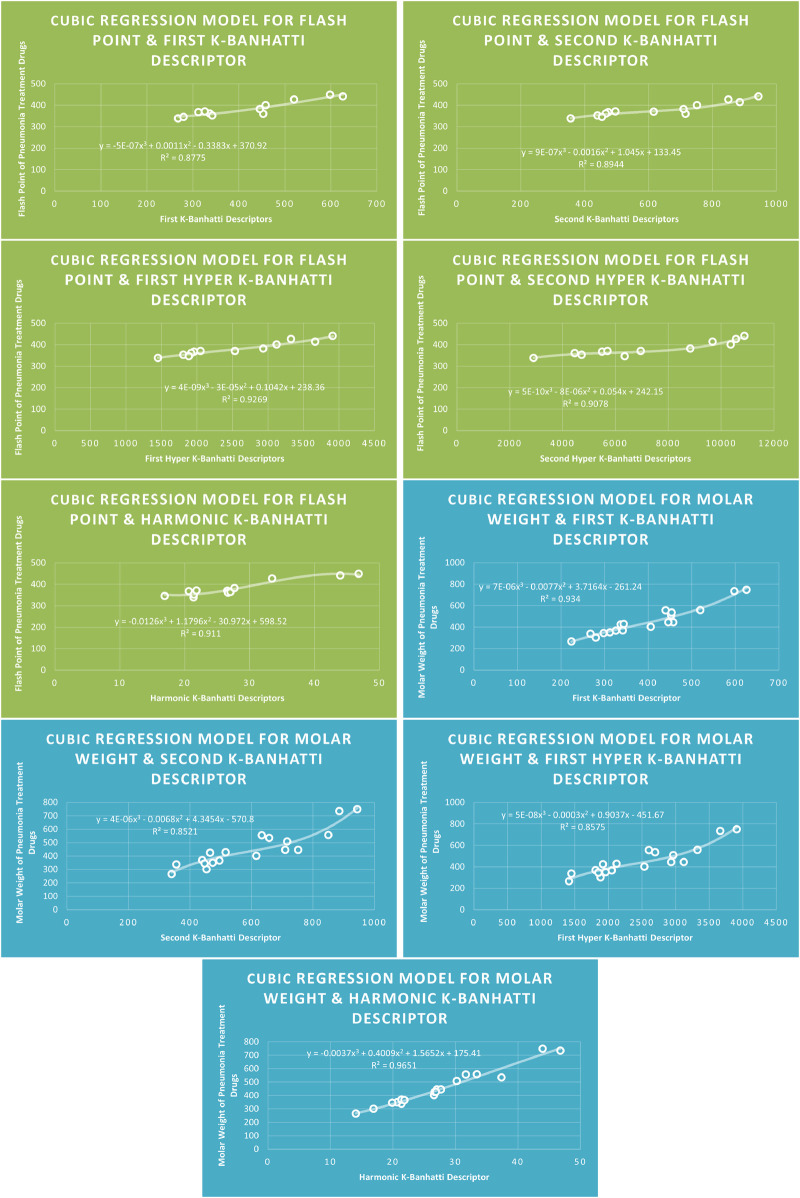
**CR** models of flash point and molar weight for pneumonia treatment drugs.

**FIGURE 12:**
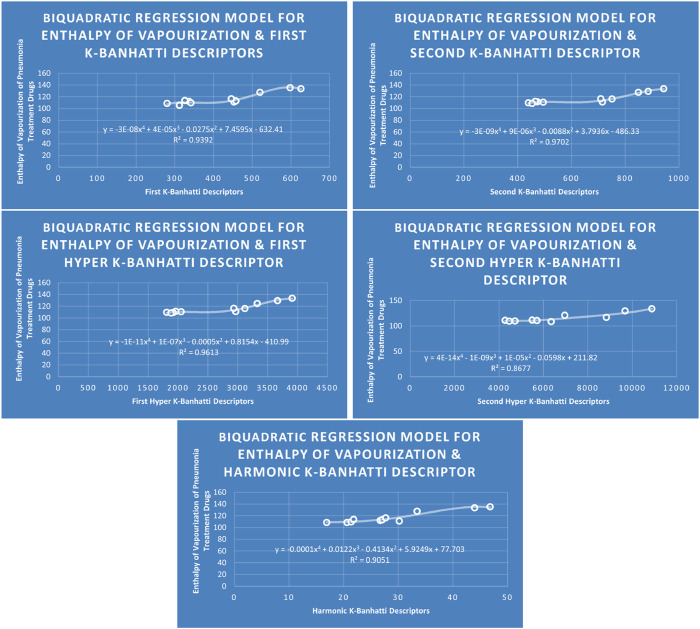
BQR models of enthalpy of vaporization for pneumonia treatment drugs.

**FIGURE 13:**
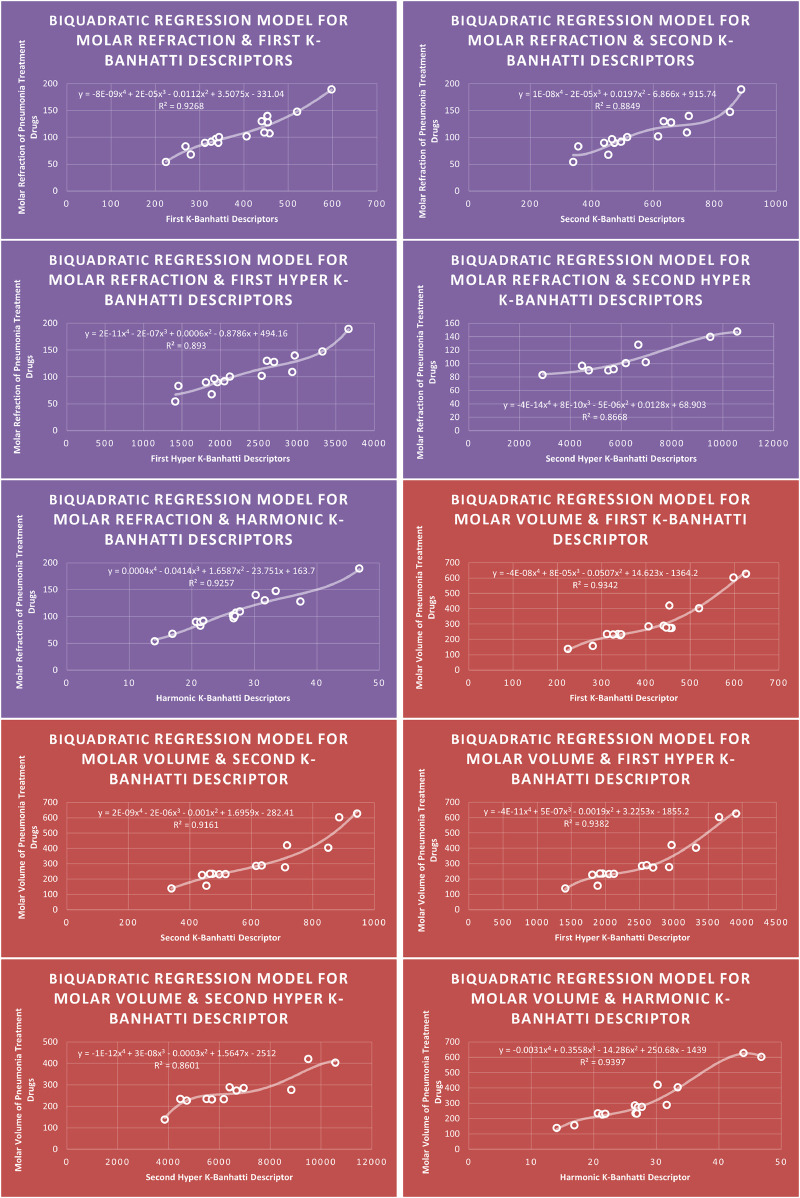
BQR models of molar refraction and molar volume for pneumonia treatment drugs.

**FIGURE 14:**
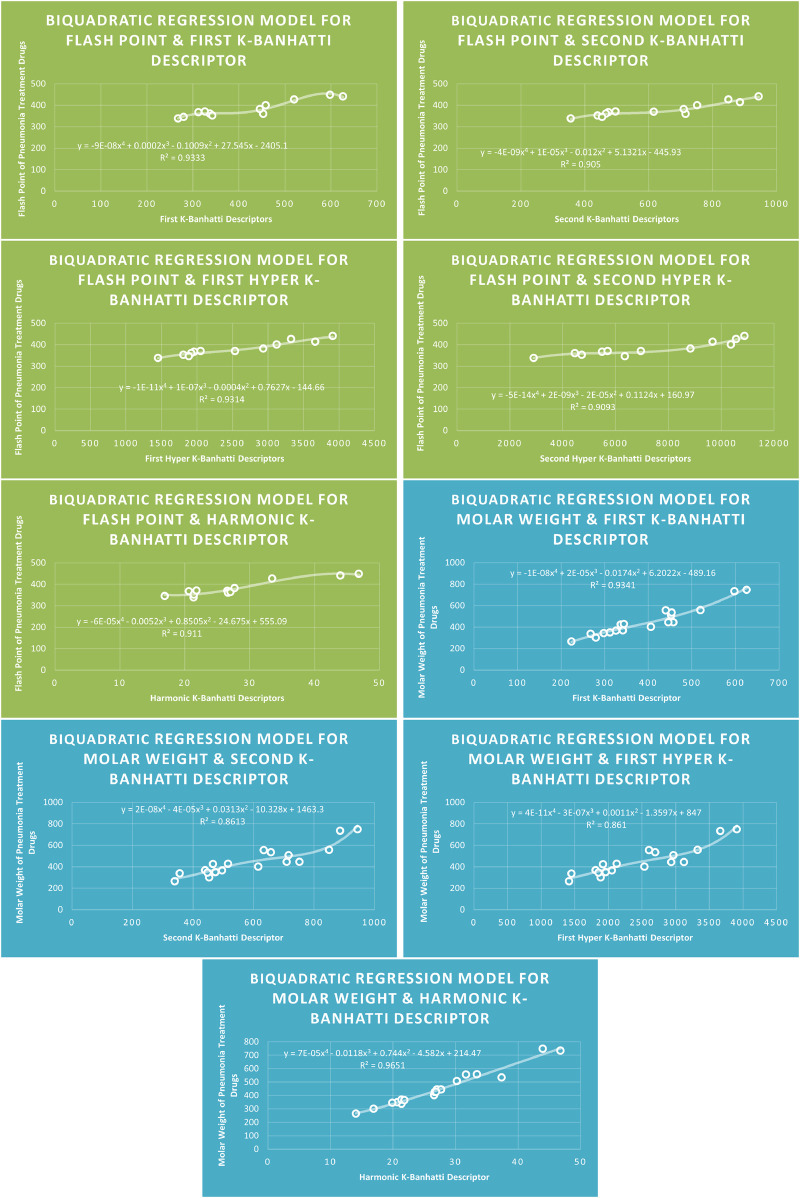
BQR models of flash point and molar weight for pneumonia treatment drugs.

The QSPR analysis and Topological descriptor exhibit a significant association, indicating a strong connection between the disease’s physical and chemical attributes. To forecast the relationship between a molecule’s structure and its behaviour or characteristics, QSPR algorithms are utilized. Several methods have been developed and used in QSPR research during the last few decades. By the use of the statistical factors ( r and $R^{2}$ ), the prediction will be verified. In real life, topological descriptors that have an actual coefficient of correlation below 0.8 are regarded as worthless. Applying linear, quadratic, cubic, and bi-quadratic regression analyses, we demonstrate the strong relationship between the attributes derived from associated topological descriptors and the physical qualities of the medications. The regression model’s quality is demonstrated by the greater $R^{2}$ value, which is very near to 1. QSPR Models Like Linear Models are shown in Figures 3–5, quadratic models are shown in Figures 6–8, cubic models are shown in Figures 9, 10, and in Figure 11 and biquadratic models are shown in Figures 12–14.

### 4.1 Linear regression analysis

The correlation between some K -Banhatti topological descriptors and the physical characteristics of different medications taken to treat pneumonia, as shown in Table 6, is obtained by employing the Linear regression framework:

$$W=\Phi \left(IN\right)+\beta$$ (6)

While W is the drug’s physical attribute ϕ is constant, β the value of the regression coefficient, and IN is the topological descriptor. Using Equation 6, the linear regression models for the given K -Banhatti topological descriptors and physicochemical properties are formulated as follows:

**TABLE 6:** Physicochemical properties of anti-pneumonia drugs.

Drugs	$E_{n}$	MW	MV	FP	MR
Linezolid	87.5 ± 3	337.346	259 ± 3	307.9 ± 30.1	83 ± 0.3
Omadacycline	127.6 ± 3	556.651	398.4 ± 5	460.4 ± 34.3	147.5 ± 0.4
Moxifloxacin	98.8 ± 3	401.431	285 ± 3	338.7 ± 31.5	101.8 ± 0.3
Ceftriaxone		554.580	281.7 ± 7		130 ± 0.5
Unasyn	105.4 ± 3	349.4	239.3 ± 5	367.4 ± 31.5	89.9 ± 4
Lefamulin	105.1 ± 6	507.726	424.8 ± 5	327.9 ± 31.5	139.8 ± 0.4
Carbapanem	74.6 ± 6	153.135	99.3 ± 5	210.9 ± 22.9	35.7 ± 0.4
Cefuroxime	112.1 ± 3	424.385	241 ± 7	396.3 ± 35.7	96.7 ± 0.5
Cefaclor	109.5 ± 3	367.807	226.5 ± 5	385.2 ± 32.9	89.6 ± 0.4
Tetracycline	113 ± 3	444.435	266.3 ± 7	400.2 ± 35.7	106.9 ± 0.5
Amoxicillin	113.7 ± 3	365.404	236.2 ± 5	403.3 ± 32.9	91.5 ± 0.4
Ceftabiprole		534.569	266.1 ± 7		127.8 ± 0.5
Doxycycline	116.5 ± 3	444.435	271.1 ± 5	415 ± 32.9	109 ± 0.4
Avibactam		265.244	143.1 ± 5		54 ± 0.4
Tazobactam	108.6 ± 3	300.291	155.8 ± 7	381.4 ± 35.7	67.7 ± 0.5
Clarithromycin	133.4 ± 6	747.953	631.9 ± 5	440.9 ± 34.3	
Levaquin		361.367	244 ± 75		91.1 ± 70.4
Erythromycin	135.4 ± 6	733.927	607.2 ± 5	448.8 ± 34.3	189.2 ± 0.4
Cefpodoxima		427.455	239.5 ± 7		100.5 ± 0.5
Penicillin	344.390				

### 4.2 Quadratic regression analysis

The correlation between some K -Banhatti topological descriptors and the physical characteristics of different medications taken to treat pneumonia as shown in Table 6, is obtained by employing the quadratic regression framework:

$$W=\Phi_{1}{\left(IN\right)}^{2}+\Phi_{2}\left(IN\right)+\beta$$ (7)

While W is the drug’s physical attributes $\left(\phi_{i},\;i=1,2\right)$ are constants, β is the value of the regression coefficient IN is the topological descriptor. Using Equation 7, the quadratic regression models for the topological descriptors given K -Banhatti and the physicochemical properties are formulated as follows:

### 4.3 Cubic regression analysis

The correlation between some K -Banhatti topological descriptors and the physical characteristics of different medications taken to treat pneumonia, as shown in Table 6, are obtained by employing the cubic regression framework:

$$W=\Phi_{1}{\left(IN\right)}^{3}+\Phi_{2}{\left(IN\right)}^{2}+\Phi_{3}\left(IN\right)+\beta$$ (8)

While W the drug’s physical attribute, $\left(\phi_{i},\;i=1,2,3\right)$ are constants, β is a constant, the value of the regression coefficient, and IN is the topological descriptor. Using Equation 8, the cubic regression models for the given K -Banhatti topological descriptors and physicochemical properties are formulated as follows:

### 4.4 Biquadratic regression analysis

The correlation between some K -Banhatti topological descriptors and the physical characteristics of different medications taken to treat pneumonia, as shown in Table 6, is obtained by employing the biquadratic regression framework:

$$W=\Phi_{1}{\left(IN\right)}^{4}+\Phi_{2}{\left(IN\right)}^{3}+\Phi_{3}{\left(IN\right)}^{2}+\Phi_{4}\left(IN\right)+\beta$$ (9)

While W is the drug’s physical attributes $\left(\phi_{i},\;i=1,2,3,4\right)$ are constants, β is the value of the regression coefficient IN is the topological descriptor. Using Equation 9, the biquadratic regression models for the given K -Banhatti topological descriptors and physicochemical properties are formulated as follows:

### 4.5 Mathematical models for linear regression

This subsection provides mathematical models obtained after incorporating QSPR analysis.

• First K -Banhatti Descriptor

$$E_{n}=0.0793\left[B_{1}\right]+83.096,\;R^{2}=0.824$$

$$MR=0.3121\left[B_{1}\right]-13.292,\;R^{2}=0.8964$$

$$MV=1.1666\left[B_{1}\right]-172.42,\;R^{2}=0.8519$$

$$MW=1.1496{\left[B_{1}\right]}_{5}.0961,\;R^{2}=0.9125$$

$$FP=0.288\left[B_{1}\right]+263.47,\;R^{2}=0.864$$

• Second K -Banhatti descriptor

$$E_{n}=0.0415\left[B_{2}\right]+89.794,\;R^{2}=0.8081$$

$$MR=0.1889{\left[B_{2}\right]}_{6}083,\;R^{2}=0.8513$$

$$MV=0.7453\left[B_{2}\right]-143.72,\;R^{2}=0.862$$

$$MW=0.6669\left[B_{2}\right]+51.895,\;R^{2}=0.8099$$

$$FP=0.1509\left[B_{2}\right]+284.34,\;R^{2}=0.8393$$

• First Hyper K -Banhatti descriptor

$$E_{n}=0.012\left[{HB}_{1}\right]+89.066,\;R^{2}=0.8203$$

$$MR=0.0472\left[{HB}_{1}\right]-3.4057,\;R^{2}=0.8571$$

$$MV=0.1843\left[{HB}_{1}\right]-155.98,\;R^{2}=0.8647$$

$$MW=0.1642\left[{HB}_{1}\right]+46.878,\;R^{2}=0.8144$$

$$FP=0.0385\left[{HB}_{1}\right]+282.61,\;R^{2}=0.9204$$

• Second Hyper K -Banhatti Descriptor

$$E_{n}=0.0035\left[{HB}_{2}\right]+92.513,\;R^{2}=0.8143$$

$$MR=0.0091\left[{HB}_{2}\right]+48.996,\;R^{2}=0.8378$$

$$MV=0.0337\left[{HB}_{2}\right]+47.06,\;R^{2}=0.8174$$

$$FP=0.011\left[{HB}_{2}\right]+300.75,\;R^{2}=0.838$$

• Harmonic K -Banhatti Descriptor

$$E_{n}=0.9878\left[H_{b}\right]+88.771,\;R^{2}=0.8719$$

$$MR=3.9318\left[H_{b}\right]+1.982,\;R^{2}=0.9204$$

$$MV=15.414{\left[H_{b}\right]}_{1}18.84,\;R^{2}=0.9012$$

$$MW=15.102\left[H_{b}\right]+35.506,\;R^{2}=0.9627$$

$$FP=3.7683\left[H_{b}\right]+275.57,\;R^{2}=0.8822$$

### 4.6 Mathematical models for quadratic regression

This subsection provides mathematical models obtained after incorporating QSPR analysis.

• First K -Banhatti Descriptor

$$E_{n}=0.0002{\left[B_{1}\right]}^{2}-0.1267\left[B_{1}\right]+126.46,\;R^{2}=0.8861$$

$$MR=0.0003{\left[B_{1}\right]}^{2}+0.0327\left[B_{1}\right]+39.615,\;R^{2}=0.9105$$

$$MV=0.0026{\left[B_{1}\right]}^{2}-1.1027\left[B_{1}\right]+283.31,\;R^{2}=0.9196$$

$$MW=0.0013{\left[B_{1}\right]}^{2}+0.0603\left[B_{1}\right]+209.54,\;R^{2}=0.9287$$

$$FP=0.0004{\left[B_{1}\right]}^{2}-0.0323\left[B_{1}\right]+329.02,\;R^{2}=0.8773$$

• Second K -Banhatti descriptor

$$E_{n}=0.0001{\left[B_{2}\right]}^{2}-0.1528\left[B_{2}\right]+149.96,\;R^{2}=0.948$$

$$MR=0.0001{\left[B_{2}\right]}^{2}+0.0479\left[B_{2}\right]+39.19,\;R^{2}=0.8596$$

$$MV=0.001{\left[B_{2}\right]}^{2}-0.5634\left[B_{2}\right]+248.5,\;R^{2}=0.902$$

$$MW=0.0006{\left[B_{2}\right]}^{2}-0.1433\left[B_{2}\right]+287.97,\;R^{2}=0.8304$$

$$FP=0.0002{\left[B_{2}\right]}^{2}-0.1206\left[B_{2}\right]+365.31,\;R^{2}=0.8735$$

• First Hyper K -Banhatti descriptor

$$E_{n}=8E-06.{\left[{HB}_{1}\right]}^{2}-0.0317\left[{HB}_{1}\right]+142.52,\;R^{2}=0.9421$$

$$MR=1E-05{\left[{HB}_{1}\right]}^{2}-0.0026\left[{HB}_{1}\right]+54.158,\;R^{2}=0.8738$$

$$MV=7E-05{\left[{HB}_{1}\right]}^{2}-0.1829\left[{HB}_{1}\right]+299.72,\;R^{2}=0.9238$$

$$MW=4E-05{\left[{HB}_{1}\right]}^{2}-0.051\left[{HB}_{1}\right]+305.14,\;R^{2}=0.8395$$

$$FP=4E-06{\left[{HB}_{1}\right]}^{2}+0.0155\left[{HB}_{1}\right]+310.75,\;R^{2}=0.9245$$

• Second Hyper K -Banhatti Descriptor

$$E_{n}=5E-07{\left[{HB}_{2}\right]}^{2}-0.0041\left[{HB}_{2}\right]+118.07,\;R^{2}=0.866$$

$$MR=6E-07{\left[{HB}_{2}\right]}^{2}+0.0012\left[{HB}_{2}\right]+73.492,\;R^{2}=0.8575$$

$$MV=2E-07{\left[{HB}_{2}\right]}^{2}+0.0314\left[{HB}_{2}\right]+54.777,\;R^{2}=0.8175$$

$$FP=1E-06{\left[{HB}_{2}\right]}^{2}-0.0059\left[{HB}_{2}\right]+354.57,\;R^{2}=0.8779$$

• Harmonic K -Banhatti Descriptor

$$E_{n}=0.0148{\left[H_{b}\right]}^{2}+0.0269\left[H_{b}\right]+102.39,\;R^{2}=0.8869$$

$$MR=-0.0035{\left[H_{b}\right]}^{2}+4.1405\left[H_{b}\right]-0.8852,\;R^{2}=0.9205$$

$$MV=0.2188{\left[H_{b}\right]}^{2}+1.9287\left[H_{b}\right]+70.546,\;R^{2}=0.9228$$

$$MW=0.0682{\left[H_{b}\right]}^{2}+10.935\left[H_{b}+93.535,\;R^{2}=0.9647\right.$$

$$FP=0.0078{\left[H_{b}\right]}^{2}+3.2651\left[H_{b}\right]+282.97,\;R^{2}=0.8825$$

### 4.7 Mathematical models for cubic regression

This subsection provides mathematical models obtained after incorporating QSPR analysis.

• First K -Banhatti Descriptor

$$E_{n}=-8E-07{\left[B_{1}\right]}^{3}+0.0013{\left[B_{1}\right]}^{2}-0.6139\left[B_{1}\right]+195.12,\;R^{2}=0.8907$$

$$MR=3E-06{\left[B_{1}\right]}^{3}-0.0038{\left[B_{1}\right]}^{2}+1.6624\left[B_{1}\right]-164.82,\;R^{2}=0.9263$$

$$MV=1E-05{\left[B_{1}\right]}^{3}-0.0114{\left[B_{1}\right]}^{2}+4.5919\left[B_{1}\right]-447.28,\;R^{2}=0.9329$$

$$MW=7E-06{\left[B_{1}\right]}^{3}-0.0077{\left[B_{1}\right]}^{2}+3.7164\left[B_{1}\right]-261.24,\;R^{2}=0.934$$

$$FP=-5E-07{\left[B_{1}\right]}^{3}+0.0011{\left[B_{1}\right]}^{2}-0.3383\left[B_{1}\right]+370.92,\;R^{2}=0.8775$$

• Second K -Banhatti descriptor

$$E_{n}=-7E-08{\left[B_{2}\right]}^{3}+0.0003{\left[B_{2}\right]}^{2}-0.246\left[B_{2}\right]+170.11,\;R^{2}=0.9483$$

$$MR=9E-07{\left[B_{2}\right]}^{3}-0.0015{\left[B_{2}\right]}^{2}+0.9698\left[B_{2}\right]-130.52,\;R^{2}=0.8702$$

$$MV=3E-06{\left[B_{2}\right]}^{3}-0.0056{\left[B_{2}\right]}^{2}+3.4581\left[B_{2}\right]-527.14,\;R^{2}=0.9159$$

$$MW=4E-06{\left[B_{2}\right]}^{3}-0.0068{\left[B_{2}\right]}^{2}+4.3454\left[B_{2}\right]-570.8,\;R^{2}=0.8521$$

$$FP=9E-07{\left[B_{2}\right]}^{3}-0.0016{\left[B_{2}\right]}^{2}+1.045\left[B_{2}\right]+133.45,\;R^{2}=0.8944$$

• First Hyper K -Banhatti descriptor

$$E_{n}=-2E-09{\left[{HB}_{1}\right]}^{3}+2E-05{\left[{HB}_{1}\right]}^{2}-0.0748\left[{HB}_{1}\right]+181.16,\;R^{2}=0.9431$$

$$MR=1E-08{\left[{HB}_{1}\right]}^{3}-9E-05{\left[{HB}_{1}\right]}^{2}+0.2312\left[{HB}_{1}\right]-123.34,\;R^{2}=0.8863$$

$$MV=4E-08{\left[{HB}_{1}\right]}^{3}-0.0003{\left[{HB}_{1}\right]}^{2}+0.6335\left[{HB}_{1}\right]-358.52,\;R^{2}=0.9338$$

$$MW=5E-08{\left[{HB}_{1}\right]}^{3}-0.0003{\left[{HB}_{1}\right]}^{2}+0.9037\left[{HB}_{1}\right]-451.67,\;R^{2}=0.8575$$

$$FP=4E-09{\left[{HB}_{1}\right]}^{3}-3E-05{\left[{HB}_{1}\right]}^{2}+0.1042\left[{HB}_{1}\right]+238.36,\;R^{2}=0.9269$$

• Second Hyper K -Banhatti Descriptor

$$E_{n}=5E-12{\left[{HB}_{2}\right]}^{3}+4E-07{\left[{HB}_{2}\right]}^{2}-0.0033\left[{HB}_{2}\right]+116.29,\;R^{2}=0.866$$

$$MR=-2E-10{\left[{HB}_{2}\right]}^{3}+5E-06{\left[{HB}_{2}\right]}^{2}-0.0235\left[{HB}_{2}\right]+118.18,\;R^{2}=0.8655$$

$$MV=1E-09{\left[{HB}_{2}\right]}^{3}-3E-05{\left[{HB}_{2}\right]}^{2}+0.2384\left[{HB}_{2}\right]-380.88,\;R^{2}=0.835$$

$$FP=5E-10{\left[{HB}_{2}\right]}^{3}-8E-06{\left[{HB}_{2}\right]}^{2}+0.054\left[{HB}_{2}\right]+242.15,\;R^{2}=0.9078$$

• Harmonic K -Banhatti Descriptor

$$E_{n}=-0.0024{\left[H_{b}\right]}^{3}+0.2388{\left[H_{b}\right]}^{2}-6.5547\left[H_{b}\right]+163.75,\;R^{2}=0.9019$$

$$MR=0.0018{\left[H_{b}\right]}^{3}-0.16785{\left[H_{b}\right]}^{2}+8.7642\left[H_{b}\right]-41.094,\;R^{2}=0.9221$$

$$MV=-0.009{\left[H_{b}\right]}^{3}+1.0287{\left[H_{b}\right]}^{2}-20.551\left[H_{b}\right]+262.4,\;R^{2}=0.9244$$

$$MW=-0.0037{\left[H_{b}\right]}^{3}+0.4009{\left[H_{b}\right]}^{2}+1.5652\left[H_{b}\right]+175.41,\;R^{2}=0.9651$$

$$FP=-0.0126{\left[H_{b}\right]}^{3}+1.1796{\left[H_{b}\right]}^{2}-30.972\left[H_{b}\right]+598.52,\;R^{2}=0.911$$

### 4.8 Mathematical models for biquadratic regression

This subsection provides mathematical models obtained after incorporating QSPR analysis.

• First K -Banhatti Descriptor

$$E_{n}=-3E-08{\left[B_{1}\right]}^{4}+4E-05{\left[B_{1}\right]}^{3}-0.0275{\left[B_{1}\right]}^{2}+7.4595\left[B_{1}\right]-632.41,\;R^{2}=0.9392$$

$$MR=-8E-09{\left[B_{1}\right]}^{4}+2E-05{\left[B_{1}\right]}^{3}-0.0112{\left[B_{1}\right]}^{2}+3.5075\left[B_{1}\right]-331.04,\;R^{2}=0.9268$$

$$MV=-4E-08{\left[B_{1}\right]}^{4}+8E-05{\left[B_{1}\right]}^{3}-0.0507{\left[B_{1}\right]}^{2}+14.623\left[B_{1}\right]-1364.2,\;R^{2}=0.9342$$

$$MW=-1E-08{\left[B_{1}\right]}^{4}+2E-05{\left[B_{1}\right]}^{3}-0.0174{\left[B_{1}\right]}^{2}+6.2022\left[B_{1}\right]-489.16,\;R^{2}=0.9341$$

$$FP=-9E-08{\left[B_{1}\right]}^{4}+0.0002{\left[B_{1}\right]}^{3}-0.1009{\left[B_{1}\right]}^{2}+27.5450\left[B_{1}\right]-2405.1,\;R^{2}=0.9333$$

• Second K -Banhatti Descriptor

$$E_{n}=-3E-09{\left[B_{2}\right]}^{4}+9E-06{\left[B_{2}\right]}^{3}-0.0O88{\left[B_{2}\right]}^{2}+3.7936\left[B_{2}\right]-486.33,\;R^{2}=0.9702$$

$$MR=1E-08{\left[B_{2}\right]}^{4}-2E-05{\left[B_{2}\right]}^{3}+0.0197{\left[B_{2}\right]}^{2}-6.866\left[B_{2}\right]+915.74,\;R^{2}=0.8849$$

$$MV=2E-09{\left[B_{2}\right]}^{4}-2E-06{\left[B_{2}\right]}^{3}-0.001{\left[B_{2}\right]}^{2}+1.6959\left[B_{2}\right]-282.41,\;R^{2}=0.9161$$

$$MW=2E-08{\left[B_{2}\right]}^{4}-4E-05{\left[B_{2}\right]}^{3}+0.0313{\left[B_{2}\right]}^{2}-10.328\left[B_{2}\right]+1463.3,\;R^{2}=0.8613$$

$$FP=-4E-09{\left[B_{2}\right]}^{4}+1E-05{\left[B_{2}\right]}^{3}-0.012{\left[B_{2}\right]}^{2}+5.1321\left[B_{2}\right]-445.93,\;R^{2}=0.905$$

• First Hyper K -Banhatti Descriptor

$$E_{n}=-1E-11{\left[{HB}_{1}\right]}^{4}+1E-07{\left[{HB}_{1}\right]}^{3}-0.0005{\left[{HB}_{1}\right]}^{2}+0.8154\left[{HB}_{1}\right]-410.99,\;R^{2}=0.9613$$

$$MR=2E-11{\left[{HB}_{1}\right]}^{4}-2E-07{\left[{HB}_{1}\right]}^{3}+0.0006{\left[{HB}_{1}\right]}^{2}-0.8786\left[{HB}_{1}\right]+494.16,\;R^{2}=0.893$$

$$MV=-4E-11{\left[{HB}_{1}\right]}^{4}+5E-07{\left[{HB}_{1}\right]}^{3}-0.0019{\left[{HB}_{1}\right]}^{2}+3.2253\left[{HB}_{1}\right]-1855.2,\;R^{2}=0.9382$$

$$MW=4E-11{\left[{HB}_{1}\right]}^{4}-3E-07{\left[{HB}_{1}\right]}^{3}+0.0011{\left[{HB}_{1}\right]}^{2}-1.3597\left[{HB}_{1}\right]+847,\;R^{2}=0.861$$

$$FP=-1E-11{\left[{HB}_{1}\right]}^{4}+1E-07{\left[{HB}_{1}\right]}^{3}-0.0004{\left[{HB}_{1}\right]}^{2}+0.7627\left[{HB}_{1}\right]-144.66,\;R^{2}=0.9314$$

• Second Hyper K -Banhatti Descriptor

$$E_{n}=4E-14{\left[{HB}_{2}\right]}^{4}-1E-09{\left[{HB}_{2}\right]}^{3}+1E-05{\left[{HB}_{2}\right]}^{2}-0.0598\left[{HB}_{2}\right]+211.82,\;R^{2}=0.8677$$

$$MR=-4E-14{\left[{HB}_{2}\right]}^{4}+8E-10{\left[{HB}_{2}\right]}^{3}-5E-06{\left[{HB}_{2}\right]}^{2}+0.0128\left[{HB}_{2}\right]+68.903,\;R^{2}=0.8668$$

$$MV=-1E-12{\left[{HB}_{2}\right]}^{4}+3E-08{\left[{HB}_{2}\right]}^{3}-0.0003{\left[{HB}_{2}\right]}^{2}+1.5647\left[{HB}_{2}\right]-2512,\;R^{2}=0.8601$$

$$FP=-5E-14{\left[{HB}_{2}\right]}^{4}+2E-09{\left[{HB}_{2}\right]}^{3}-2E-05{\left[{HB}_{2}\right]}^{2}+0.1124\left[{HB}_{2}\right]+160.97,\;R^{2}=0.9093$$

• Harmonic K -Banhatti Descriptor

$$E_{n}=-0.0001{\left[H_{b}\right]}^{4}+0.0122{\left[H_{b}\right]}^{3}-0.4134{\left[H_{b}\right]}^{2}+5.9249\left[H_{b}\right]+77.703,\;R^{2}=0.9051$$

$$MR=0.0004{\left[H_{b}\right]}^{4}-0.0414{\left[H_{b}\right]}^{3}+1.6587{\left[H_{b}\right]}^{2}-23.751\left[H_{b}\right]+163.7,\;R^{2}=0.9257$$

$$MV=-0.0031{\left[{HB}_{1}\right]}^{4}+0.3558{\left[{HB}_{1}\right]}^{3}-14.286{\left[{HB}_{1}\right]}^{2}+250.68\left[{HB}_{1}\right]-1439,\;R^{2}=0.9397$$

$$MW=7E-05{\left[H_{b}\right]}^{4}-0.0118{\left[H_{b}\right]}^{3}+0.744{\left[H_{b}\right]}^{2}-4.582\left[H_{b}\right]+214.47,\;R^{2}=0.9651$$

$$FP=-6E-05{\left[H_{b}\right]}^{4}-0.0052{\left[H_{b}\right]}^{3}+0.8505{\left[H_{b}\right]}^{2}-24.675\left[H_{b}\right]+555.09,\;R^{2}=0.911$$

## 5 Discussions

The correlation evaluation specifies the proportion of the connection and offers additional details regarding the association of parameters. Squaring the correlation coefficient yields the correlation of determination $\left(R^{2}\right)$ . In Table 7 correlation coefficients are given. The ${HB}_{2}\left(G\right)$ descriptor for molecular weight is not best fitted for any model because for this desriptor correlation value is below 0.8. The $B_{2}\left(G\right)$ descriptor provides the highest correlation coefficient for Enthalpy of vaporization (r=0.9850) for Biquadratic regression, $B_{1}\left(G\right)$ descriptor provides the highest correlation coefficient for molar Refraction (r=0.9627) for biquadratic regression, $H_{b}\left(G\right)$ descriptor provides the highest correlation coefficient for molar volume (r=0.9694) for biquadratic regression. The $B_{1}\left(G\right)$ descriptor has the strongest correlation coefficient for flash point (0.9661) for Biquadratic regression, and $H_{b}\left(G\right)$ the descriptor provides the highest correlation coefficient (r=0.9824) for Cubic and Biquadratic regression. Moreover, Table 7 highlights the highest correlation value against each property and descriptor for every model for ready reference.

**TABLE 7:** Correlation coefficient ( R ) of physicochemical properties for Linear, Quadratic, Cubic, and Biquadratic regression model for K Banhatti Descriptors.

Model	Descriptor	$E_{n}$	MR	MV	FP	MW
Linear	$B_{1}$	0.9077	0.9468	0.9230	0.9295	0.9552
	$B_{2}$	0.8989	0.9227	0.9284	0.9161	0.8999
	${HB}_{1}$	0.9057	0.9258	0.9299	0.9594	0.9024
	${HB}_{2}$	0.9024	0.9153	0.9041	0.9154	
	$H_{b}$	0.9338	0.9594	0.9493	0.9393	0.9812
	$B_{1}$	0.9413	0.9542	0.9590	0.9366	0.9637
	$B_{2}$	0.9737	0.9271	0.9497	0.9346	0.9113
Quadratic	${HB}_{1}$	0.9706	0.9348	0.9611	0.9615	0.9162
	${HB}_{2}$	0.9306	0.9260	0.9042	0.9370	
	$H_{b}$	0.9418	0.9594	0.9606	0.9394	0.9822
	$B_{1}$	0.9438	0.9624	0.9659	0.9367	0.9664
	$B_{2}$	0.9738	0.9328	0.9570	0.9457	0.9231
Cubic	${HB}_{1}$	0.9711	0.9414	0.9663	0.9628	0.9260
	${HB}_{2}$	0.9306	0.9303	0.9138	0.9528	
	$H_{b}$	0.96497	0.9603	0.9615	0.9545	0.9824
	$B_{1}$	0.9691	0.9627	0.9665	0.9661	0.9665
	$B_{2}$	0.9850	0.9407	0.9571	0.9513	0.9281
Biquadratic	${HB}_{1}$	0.9805	0.9450	0.9686	0.9651	0.9279
	${HB}_{2}$	0.9315	0.9310	0.9274	0.9536	
	$H_{b}$	0.9514	0.9621	0.9694	0.9544	0.9824

## 6 Concluding remarks

This research employs K -Banhatti topological descriptors derived from pharmaceutical chemical graphs to develop QSPR models for pneumonia therapeutics. The testing of our model shows that it can make predictions, which suggests that it could be a useful tool for guiding the research into drug therapies for pneumonia. In this paper, the K -Banhatti descriptors were computed. We utilized the quantitative method by expanding the K -Banhatti topological descriptors for the estimation of the physicochemical properties of medications for pneumonia. This work employed linear, quadratic, cubic, and biquadratic regressions to evaluate the relationship between properties and the K -Banhatti descriptors. The correlation between physicochemical properties and our findings is represented in Table 7, which implies that every model for properties was determined to be valid and provide a good correlation. The findings give an economical and scientific basis for developing novel medicines having comparable designs for greater effect and therapy. The study suggests that pharmacists and scientists can build anti-pneumonia medications based on such topological descriptors. We have discovered that topological descriptors have a correlation coefficient, suggesting that we can combine medications with an elevated correlation to create novel medications. Below are some of the best approximations from this study.

• Best approximated result for Linear, Quadratic, Cubic, and Biquadratic regression for Enthalpy of vaporization

$$E_{n}=0.9878\left[H_{b}\right]+88.771,\;R^{2}=0.8719$$

$$E_{n}=0.0001{\left[B_{2}\right]}^{2}-0.1528\left[B_{2}\right]+149.96,\;R^{2}=0.948$$

$$E_{n}=-7E-08{\left[B_{2}\right]}^{3}+0.0003{\left[B_{2}\right]}^{2}-0.246\left[B_{2}\right]+170.11,\;R^{2}=0.9483$$

$$E_{n}=-3E-09{\left[B_{2}\right]}^{4}+9E-06{\left[B_{2}\right]}^{3}-0.0088{\left[B_{2}\right]}^{2}+3.7936\left[B_{2}\right]-486.33,\;R^{2}=0.9702$$

• Best approximated result for Linear, Quadratic, Cubic, and Biquadratic regression for Flash point

$$FP=0.0385\left[{HB}_{1}\right]+282.61,\;R^{2}=0.9204$$

$$FP=4E-06{\left[{HB}_{1}\right]}^{2}+0.0155\left[{HB}_{1}\right]+310.75,\;R^{2}=0.9245$$

$$FP=4E-09{\left[{HB}_{1}\right]}^{3}-3E-05{\left[{HB}_{1}\right]}^{2}+0.1042\left[{HB}_{1}\right]+238.36,\;R^{2}=0.9269$$

$$FP=-9E-08{\left[B_{1}\right]}^{4}+0.0002{\left[B_{1}\right]}^{3}-0.1009{\left[B_{1}\right]}^{2}+27.5450\left[B_{1}\right]-2405.1,\;R^{2}=0.9333$$

• Best approximated result for Linear, Quadratic, Cubic, and Biquadratic regression for Molar refraction

$$MR=3.9318\left[H_{b}\right]+1.982,\;R^{2}=0.9204$$

$$MR=-0.0035{\left[H_{b}\right]}^{2}+4.1405\left[H_{b}\right]-0.8852,\;R^{2}=0.9205$$

$$MR=3E-06{\left[B_{1}\right]}^{3}-0.0038{\left[B_{1}\right]}^{2}+1.6624\left[B_{1}\right]-164.82,\;R^{2}=0.9263$$

$$MR=-8E-09{\left[B_{1}\right]}^{4}+2E-05{\left[B_{1}\right]}^{3}-0.0112{\left[B_{1}\right]}^{2}+3.5075\left[B_{1}\right]-331.04,\;R^{2}=0.9268$$

• Best approximated result for Linear, Quadratic, Cubic, and Biquadratic regression for Molar volume

$$MV=15.414{\left[H_{b}\right]}_{1}18.84,\;R^{2}=0.9012$$

$$MV=7E-05{\left[{HB}_{1}\right]}^{2}-0.1829\left[{HB}_{1}\right]+299.72,\;R^{2}=0.9238$$

$$MV=4E-08{\left[{HB}_{1}\right]}^{3}-0.0003{\left[{HB}_{1}\right]}^{2}+0.6335\left[{HB}_{1}\right]-358.52,\;R^{2}=0.9338$$

$$MV=-4E-11{\left[{HB}_{1}\right]}^{4}+5E-07{\left[{HB}_{1}\right]}^{3}-0.0019{\left[{HB}_{1}\right]}^{2}+3.2253\left[{HB}_{1}\right]-1855.2,\;R^{2}=0.9382$$

• Best approximated result for Linear, Quadratic, Cubic, and Biquadratic regression for Molar weight

$$MW=15.102\left[H_{b}\right]+35.506,\;R^{2}=0.9627$$

$$MW=0.0682{\left[H_{b}\right]}^{2}+10.935\left[H_{b}+93.535,\;R^{2}=0.9647\right.$$

$$MW=-0.0037{\left[H_{b}\right]}^{3}+0.4009{\left[H_{b}\right]}^{2}+1.5652\left[H_{b}\right]+175.41,\;R^{2}=0.9651$$

$$MW=7E-05{\left[H_{b}\right]}^{4}-0.0118{\left[H_{b}\right]}^{3}+0.744{\left[H_{b}\right]}^{2}-4.582\left[H_{b}\right]+214.47,\;R^{2}=0.9651$$

For this study, the cubic and Biquadratic regressions give more reliable results as compared to linear and quadratic. From these models and Table 7, we have oder of reliability:

Linear<Quadratic<Cubic<Biquadratic

## Data Availability

The original contributions presented in the study are included in the article/supplementary material, further inquiries can be directed to the corresponding authors.
